# Effectiveness, User Engagement and Experience, and Safety of a Mobile App (Lumi Nova) Delivering Exposure-Based Cognitive Behavioral Therapy Strategies to Manage Anxiety in Children via Immersive Gaming Technology: Preliminary Evaluation Study

**DOI:** 10.2196/29008

**Published:** 2022-01-24

**Authors:** Joanna Lockwood, Laura Williams, Jennifer L Martin, Manjul Rathee, Claire Hill

**Affiliations:** 1 National Institute of Health Research MindTech MedTech Co-operative School of Medicine University of Nottingham Nottingham United Kingdom; 2 BFB Labs Ltd London United Kingdom; 3 School of Psychology & Clinical Language Sciences University of Reading Reading United Kingdom

**Keywords:** anxiety, children, exposure therapy, cognitive behavioral therapy, immersive gaming, digital intervention, app, smartphone, mobile phone

## Abstract

**Background:**

Childhood anxiety disorders are a prevalent mental health problem that can be treated effectively with cognitive behavioral therapy, in which exposure is a key component; however, access to treatment is poor. Mobile-based apps on smartphones or tablets may facilitate the delivery of evidence-based therapy for child anxiety, thereby overcoming the access and engagement barriers of traditional treatment. Apps that deliver therapeutic content via immersive gaming technology could offer an effective, highly engaging, and flexible treatment proposition.

**Objective:**

In this paper, we aim to describe a preliminary multi-method evaluation of Lumi Nova, a mobile app intervention targeting mild to moderate anxiety problems in children aged 7-12 years using exposure therapy delivered via an immersive game. The primary objective is to evaluate the effectiveness, user engagement and experience, and safety of the beta version of Lumi Nova.

**Methods:**

Lumi Nova was co-designed with children, parents, teachers, clinicians, game industry experts, and academic partnerships. In total, 120 community-based children with mild to moderate anxiety and their guardians were enrolled to participate in an 8-week pilot study. The outcome measures captured the app’s effectiveness (anxiety symptoms, child-identified goal-based outcomes, and functional impairment), user engagement (game play data and ease-of-use ratings), and safety (mood ratings and adverse events). The outcome measures before and after the intervention were available for 30 children (age: mean 9.8, SD 1.7 years; girls: 18/30, 60%; White: 24/30, 80%). Additional game play data were automatically generated for 67 children (age: mean 9.6, SD 1.53 years; girls: 35/67, 52%; White: 42/67, 63%). Postintervention open-response data from 53% (16/30) of guardians relating to the primary objectives were also examined.

**Results:**

Playing Lumi Nova was effective in reducing anxiety symptom severity over the 8-week period of game play (*t*_29_=2.79; *P*=.009; Cohen *d*=0.35) and making progress toward treatment goals (*z*=2.43; *P*=.02), but there were no improvements in relation to functional impairment. Children found it easy to play the game and engaged safely with therapeutic content. However, the positive effects were small, and there were limitations to the game play data.

**Conclusions:**

This preliminary study provides initial evidence that an immersive mobile game app may safely benefit children experiencing mild to moderate anxiety. It also demonstrates the value of the rigorous evaluation of digital interventions during the development process to rapidly improve readiness for full market launch.

## Introduction

### Background

Anxiety disorders are among the most common and impairing mental health difficulties experienced in childhood and are characterized by excessive fear, worry, and negative beliefs that can result in distress and functional impairments in social, academic, and family life [1,2]. Anxiety disorders typically begin in childhood [3], often co-occurring with other anxiety disorders and depressive, behavioral, and neurodevelopmental disorders [4]. When not treated successfully, children who experience high levels of anxiety can continue to have problems over their life course and are at increased risk for other persistent, long-term adverse outcomes [2,4-6]. Recent national survey data from the United Kingdom indicate that emotional difficulties (anxiety and low mood) have increased by nearly 50% in young people over the 2004-2017 period [1]. Into what was already a concerning situation, the COVID-19 pandemic has contributed significant disruption and uncertainty to young lives, and early findings point to heightening anxiety in primary-age children and those with pre-existing vulnerabilities during the first stages of lockdown [7]. Early identification and access to effective treatment is critical.

### Evidence-Based Treatment and Associated Challenges

Substantial clinical evidence suggests that anxiety in children can be effectively treated using psychological approaches [8]. Cognitive behavioral therapy (CBT) demonstrates consistent superiority in randomized controlled trials over no therapy for mild to moderate childhood anxiety, and consequently is the first-line recommended treatment for children and young people [9,10]. CBT for anxiety involves psychoeducation, identifying and challenging anxious thoughts, facing feared objects and situations through graded exposure, and problem-solving techniques. Treatment format, delivery in shortened form, or comorbidity does not appear to substantially alter CBT efficacy [9,11]. However, around a third of children and adolescents retain their primary anxiety disorder following a course of CBT treatment, suggesting that alternative or more targeted approaches are warranted [11]. Most children who could benefit from an intervention do not access formal support. Around 60% of children with anxiety disorders do not seek professional help, with only a small minority receiving support from specialist mental health services (15.2%), and less than 3% receiving CBT [12,13]. Barriers include overstretched mental health services with lengthy waitlists, as well as attitudinal issues around stigma, negative beliefs or lack of awareness about mental health services, and preferences for self-help over clinical support [14-16]. Poor adherence to treatment and high dropout rates (23%-60%) are also a threat to treatment benefits and suggest that the interventions may be lacking appeal for young people [17,18].

Importantly, although expert consensus and dismantling studies indicate that exposure-based elements of CBT are active components that are effective in treating anxiety disorders, exposure-based CBT is infrequently included in interventions for children [19,20]. This underutilization may relate to high costs and time constraints, as well as a lack of therapist training and confidence or negative beliefs about the approach [21,22]. In particular, anxious children may lack intrinsic motivation to comply with exposure elements of therapy, given that they are unlikely to have initiated help-seeking in the first place and they might be naturally hesitant to face anxiety-provoking situations [22]. Novel treatment platforms for therapy delivery that (1) appeal to children, (2) are accessible to children, and (3) optimize exposure-based treatment in ways that are acceptable to children may help address some of the barriers to successful treatment.

### Maximizing Access and Benefit Through Mobile Apps

Digital mental health interventions (including web-based or computer-based programs) that draw on CBT-based techniques are effective in reducing anxiety symptom severity in children and young people [23-26]. However, the evidence base is limited, particularly for younger children [25], the uptake and adherence to treatment for young people is often low or variable, dropout rates can be high, and there are little systematic data on levels of engagement. Outside of controlled clinical trials, real-world uptake and adherence with web-based digital interventions for mood disorders are similarly variable [27], which makes it difficult to establish the translation of impact to natural settings. The evaluation of digital interventions is largely limited to web-based or computer-based programs that were developed several years ago and, crucially, did not draw on a co-design approach. Recent guidance has called for increased participatory approaches that actively engage stakeholders throughout the development cycle of digital interventions to ensure that innovations fit needs, are acceptable, and are used [28]. Transparent reporting of the contribution of co-design and user-centered processes is necessary to benchmark their role in the development of new innovations [29-31].

Outcomes may be optimized for children when the capabilities of mobile technologies (smartphones and tablets) are fully leveraged. Many children are comfortable and familiar with processing information and engaging with content via mobile devices. The levels of digital independence for children are increasing, with around 50% of those aged 8-11 years using a smartphone and 72% using a tablet [32]. Interventions delivered remotely via mobile devices (mobile health [mHealth]) may bring the advantage of increased appeal and access for young people, potentially extending to those less likely to access support in traditional mental health settings [33]. However, although mHealth interventions may hold promise [34], few child-focused interventions have been subject to empirical evaluation [35-37] or are supported only in relation to feasibility, but not efficacy, usability, or safety [36,38,39]. Given the increasing ubiquity of apps for childhood mental health, robust evaluation studies are a research priority. Recent guidance has called for granular evaluation of use and engagement indicators in mHealth apps, including multidimensional objective and subjective engagement measures, and to understand how apps impact treatment outcomes [40,41].

### Immersive Games for Anxiety

The application of game design elements is heralded as a strategy to increase engagement and adherence with mHealth interventions, offering an intrinsically motivating option for therapeutic delivery for children, particularly where content supports user preferences for being interactive, personable, and relatable [30,42]. Although empirical evidence to support game-based mental health interventions for childhood anxiety is lacking [29,33], increased user engagement and improved outcomes have been attributed to the integration of gamification techniques and interactive features in smartphone-delivered CBT [43]. The gamification elements that scaffold learning may help to make complex models of therapy (such as CBT) more understandable for children [44]. The structured stepped approach in exposure therapy is also suited to a game format in which progression and reward systems lend themselves to graduated challenges and motivation. Digital innovation offers the potential to deliver exposure-based therapy through immersive technologies (eg, providing the user with an experience of being able to view and interact with simulated objects and environments such as 360-degree photography and virtual and augmented reality). This innovation may help overcome some of the practical and cost barriers to delivering exposure therapy in real-world settings. Limited data support the viability of using computer games and video-based platforms to support the delivery of CBT-based therapeutic processes, including exposure tasks for childhood mental health problems [44-46], and studies have shown that exposure-based game mechanics provide an effective therapeutic action mechanism [47]. However, robust outcome evidence is sparse, and high-end immersive game-based apps that deliver structured exposure-based treatment at a self-help level for children remain underexplored.

### Study Objectives

This preliminary study evaluates the effectiveness, user engagement and experience, and safety of a novel app for smartphones and tablets (Lumi Nova), which uses immersive gaming technology to deliver exposure therapy for children aged 7-12 years with mild to moderate anxiety difficulties. Specifically, the primary objective is to evaluate the following: (1) whether exposure therapy delivered via Lumi Nova is associated with a reduction in guardian-reported anxiety symptoms and functional impairment in children and progression toward the child-identified goals related to anxiety; (2) user engagement, ease of use, and experience of Lumi Nova; and (3) whether Lumi Nova is safe to use (ie, is not associated with harm or unintended negative consequences). Our expectation is that playing Lumi Nova would be associated with lower anxiety symptom severity and interference after the intervention and positive progression toward treatment goals. No further hypotheses have been offered for this exploratory study.

## Methods

### Study Design

Multiple quantitative and qualitative methods were used. A pre-post design was used to compare the guardian-rated outcome measures captured via survey before (T1) and after the intervention (T2). In addition, game play data were collected over the course of the intervention, and player ratings and guardian open survey responses were collected after the intervention (T2). Data were collected during a 10-week intervention period between January and March 2020 with game play data generated over approximately 8 weeks of play. The study was approved by the Faculty of Medicine and Health Sciences Research Ethics Committee, University of Nottingham (Reference: 452-1911; December 19, 2019).

### Participants

A total of 120 English-speaking children aged 7-12 years and their guardians completed T1 anxiety measures. Children were identified by school-based staff in 12 participating schools as experiencing difficulties with anxiety and not concurrently receiving psychological treatment. The participating schools were 9 primary schools and 3 secondary schools in the South East England identified through a partnership with the local council Personal, Social, Health and Economic education curriculum and Healthy School Lead and supported by a Children and Young People’s Mental Health and Wellbeing Steering Group. The mean eligibility for free school meals across these schools (a proxy for socioeconomic status) was 18.1% (SD 7.3%), indicating that the school sample from which children were drawn was broadly representative of nationally reported proportions (15.8%) across all primary school types (Department for Education, January 2019). Most children (88/120, 73.3%) had not sought or received previous treatment for anxiety before starting the pilot intervention through the Children and Adolescent Mental Health Service (CAMHS), a general practitioner or nurse (92/120, 76.7%), or a psychologist or counselor (94/120, 78.3%).

**Figure 1:**
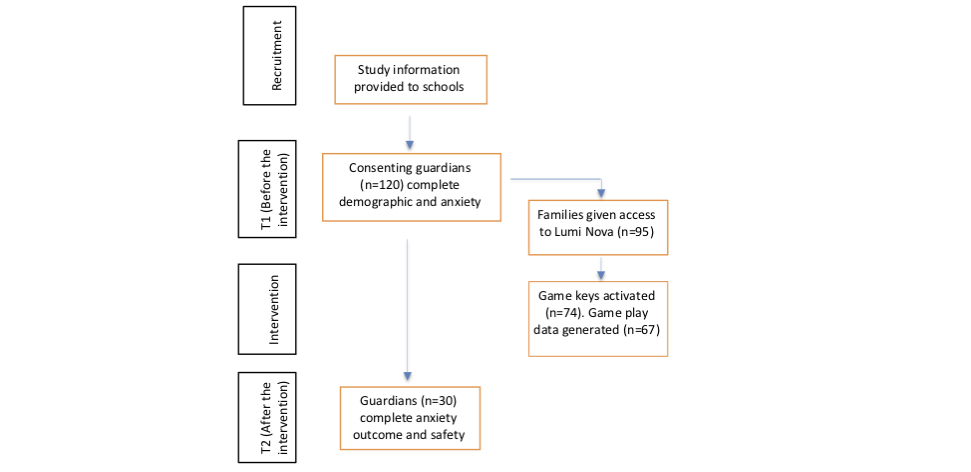
Overview of the recruitment and study process.

Of the 120 participants with complete anxiety-related outcome measures at T1, follow-up measures at T2 were available for 30 (25%) children aged 6-13 years (mean 9.8, SD 1.7 years); 2 (1.7%) children were marginally outside the target age range of 7-12 years (aged 6.97 and 13.0 years) at the point of entering the study and were retained in the analysis. Of the 120 guardians from the T1 sample, 95 (79.2%) completed an additional anxiety measure survey following an automated SMS text message prompt to be provided with a game key, and 74 (61.7%) guardians activated the game key and downloaded Lumi Nova. Subsequent game play data were recorded for 67 (71%) out of 95 participants. Among the 30 participants with complete T1-T2 anxiety-related outcome measures, game play data were recorded for 25 (83%). Details of the study recruitment and attrition are shown in Figure 1.

Analyses were conducted on the two subsamples for whom there was complete data: the T1-T2 complete outcome measure subsample (n=30) and the game play analytics subsample (n=67). The demographic characteristics and outcome variables of these samples are presented in Table 1. Children for whom there were complete outcome measures at T1 and T2 did not differ statistically on demographic variables or outcome measures (based on 2-tailed independent samples *t* tests and chi-square tests) before the intervention in comparison to the 90 children lost to follow-up at T2: gender (*P*=.32), ethnicity (*P*=.12), disability (*P*=.76), free school meal status (*P*=.22), predominant language (*P*=.32), other anxiety treatment (*P*=.06), Revised Child Anxiety and Depression Scale–Parent version (RCADS-P; *P*=.052), Child Anxiety Impact Scale–Parent version (CAIS-P; *P*=.33); however, they were significantly more anxious (*P*=.04; Spence Child Anxiety Scale–Parent version [SCAS-P-8]). Children who played Lumi Nova for whom we had complete outcome measures (25/67, 37%) did not statistically differ from those who played the game but did not provide outcome measure data (42/67, 63%) on demographic variables or outcome measures before the intervention. Regarding clinical characteristics, before the intervention, 40% (12/30) of the T1-T2 subsample and 23% (15/67) of the game play subsample scored within a clinical range for anxiety disorders.

**Table 1:** Demographic data and clinical characteristics for study subsamples.

Demographic details			T1-T2 subsample (n=30)	Game play subsample (n=67)
Age^a^ (years), mean (SD)			9.81 (1.70)	9.6 (1.53)
**Gender, n (%)**				
	Male		12 (40)	31 (46)
	Female		18 (60)	35 (52)
**Free school meals, n (%)**				
	Yes		10 (33)	17 (25)
**Disability, n (%)**				
	No		29 (97)	64 (96)
**Ethnicity, n (%)**				
	Asian or Asian British		1 (3)	2 (3)
	Black or African or Caribbean or Black British		3 (10)	8 (12)
	Mixed or multiple ethnicities		1 (3)	4 (6)
	Other ethnic groups		1 (3)	1 (1)
	White		24 (80)	42 (63)
**Predominant language, n (%)**				
	English		30 (100)	57 (85)
**Treatment history^b^, n (%)**				
	**Other anxiety treatment**			
		No	25 (83)	53 (79)
		Yes	2 (7)	3 (4)
		Do not know	1 (3)	1 (1)
	**CAMHS^c^** **contact for anxiety**			
		No	23 (77)	46 (69)
		Yes	6 (20)	10 (15)
		Do not know	1 (3)	1 (1)
	**GP^d^** **or nurse contact for anxiety**			
		No	25 (83)	48 (72)
		Yes	5 (16)	8 (12)
		Do not know	0 (0)	1 (1)
**Clinical characteristics (n=59), mean (SD)**				
	SCAS-P-8^e^		8.33 (4.56)	7.83 (3.71)
	RCADS-P^f,g^ (total anxiety)		30.30 (16.92)	28.97 (14.45)
	CAIS-P^h^ (total)		20.57 (15.40)	18.39 (13.25)
**Clinical thresholds^i,j,k^ (n=56), n (%)**				
	At clinical cutoff		8 (40)	13 (23)
	At borderline cutoff		1 (5)	4 (7)
	Within normal range		11 (55)	39 (70)

^a^Game play subsample age was based on 59 responses.

^b^Treatment history was based on the previous 3 months.

^c^CAMHS: Children and Adolescent Mental Health Service.

^d^GP: general practitioner.

^e^SCAS-P-8: Spence Child Anxiety Scale–Parent version.

^f^RCADS-P: Revised Child Anxiety and Depression Scale–Parent version.

^g^Clinical characteristics were based on 58 responses for the Revised Child Anxiety and Depression Scale–Parent version.

^h^CAIS-P: Child Anxiety Impact Scale–Parent version.

^i^Clinical thresholds describe the top 2% of scores of unreferred children of the same age and the top 7% for borderline clinical threshold.

^j^Clinical cutoffs were based on 56 participants who met the age range for standardized Revised Child Anxiety and Depression Scale–Parent version *t* scores (*t* scores are calculated from raw scores to enable comparison of anxiety scores to population-level data).

^k^For the T1-T2 subsample, the clinical cutoffs were based on 20 participants who met the age range for standardized Revised Child Anxiety and Depression Scale–Parent version *t* scores.

### Intervention Development and Therapeutic Approach

Lumi Nova combines evidence-based therapeutic content (exposure therapy) and psychoeducational content within an immersive game designed to provide timely support to children aged 7-12 years, who are facing difficulties with anxiety. The app uses a diverse range of techniques, including storytelling, photographs, videos, 360° videos, and game mechanics with a progressive narrative, rewards, customization of avatars, and unlocking new levels to deliver an immersive experience to users. The development and design of Lumi Nova resulted from a robust coproduced and collaborative user-centered design process that involved children, parents, teachers, clinical practitioners, academics, and game industry experts to build the game concept, design, and clinical model parameters. In the initial phase of development, the aim was to develop a prototype game that delivered exposure therapy in a way that would be engaging, effective, and viable for children. The development phase involved multiple and multi-school site cocreation and user-testing sessions, and early prototype testing sessions with key stakeholders over a period of 5 months.

**Figure 2:**
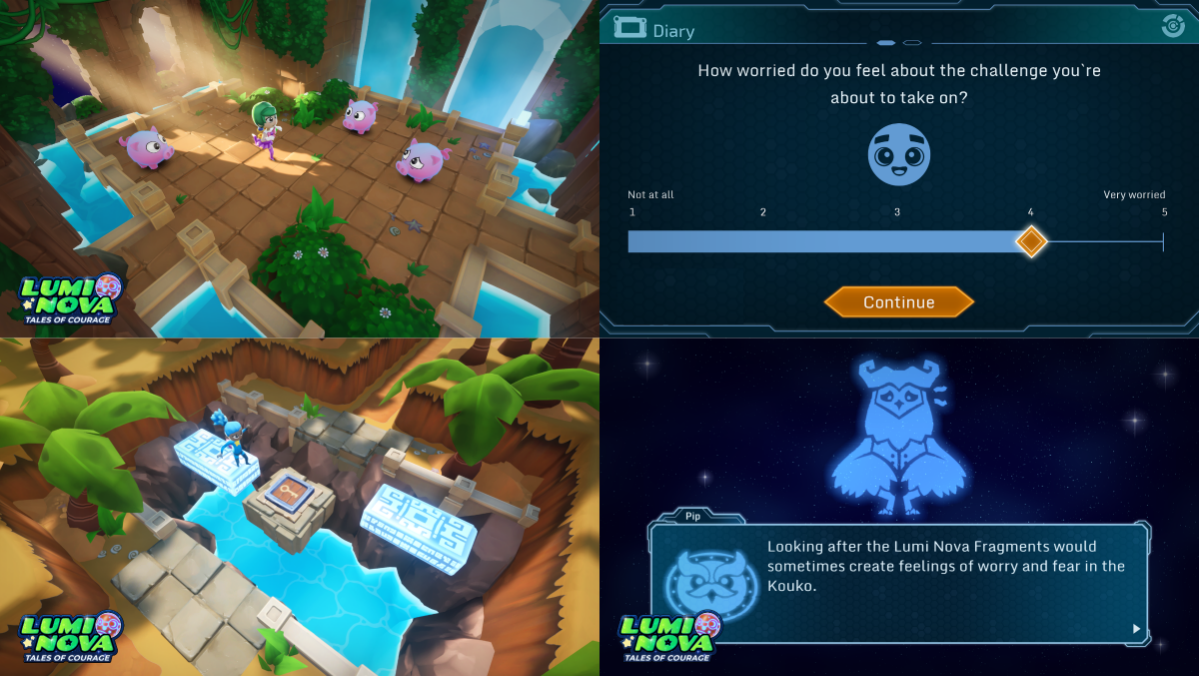
Example screenshots from Lumi Nova game play.

The game narrative is an intergalactic role-playing adventure in which players assume the role of a treasure hunter on a quest to save the galaxy and explore the universe, helping characters on various planets while training to overcome real-world fears (Figure 2). The game is played independently and is downloadable to a mobile or tablet (Android [Google Inc], and iOS [Apple Inc]) and does not require additional hardware or software. Guardians are the parents or carers, or other adults with parental responsibility. Guardian involvement is encouraged through automated SMS text message technology triggered by the child’s progress in the game and is necessary for goal-setting and the supervision of out-of-game challenges.

**Figure 3:**
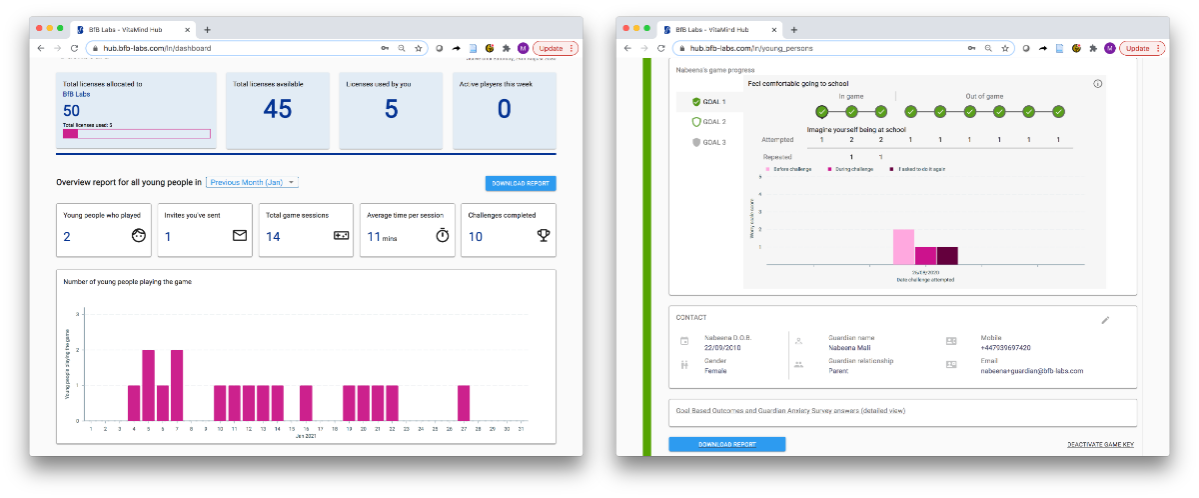
Example screenshots of VitaMind Hub. Progress data are accessible to authorized professionals to facilitate active remote monitoring and care decisions.

The mechanics of the intervention facilitates players to set anxiety-related goals and build a graded ladder of exposure steps (challenges) and to undertake these steps recording their before, after, and future exposure reflection in response to clinical psychologist determined prompts (eg, “What do you think might happen during this challenge?” “How worried did you feel during the challenge?” “How worried would you feel if you have to do it again?”). This approach is underpinned by strategies for optimizing learning during exposure, based on inhibitory learning perspectives. Negative expectancies associated with a perceived aversive outcome are countered by emphasizing the mismatch between what is expected to occur and what actually occurs [48]. In total, 14 common anxiety-related goals are available for selection during game play, which are related to social anxiety, separation anxiety, and specific phobias. Anxiety goals and exposure steps were determined in consultation with clinicians, parents, and children during coproduction workshops. These include exposure steps completed within the game, and within a real-world setting (in vivo) with guardian support, to combine multiple opportunities and varied contexts for exposure practice in line with recommended practice [48]. Players must complete each exposure step to progress through the game and achieve their goals. The game also provides embedded psychoeducational information about anxiety and exposure therapy. There was no suggested amount of time for game play per session. However, players can only play for up to 40 minutes per day after the first session, which included a tutorial. This time limit was judged as providing sufficient time to engage beneficially with Lumi Nova but was short enough to address parental concerns around too much screen time [49]. Access to Lumi Nova is provided through a secure web-based platform, VitaMind Hub (BfB Labs Ltd), which is a point of access for professionals and tracks player progress with the game (Figure 3). Progress data included the goals the child was working on, child worry scales before and after each challenge step, child-reported progress toward reaching their goal (ie, goal-based outcomes [GBOs]), and scores on a brief guardian-reported anxiety measure (ie, SCAS-P-8) before the intervention and after the completion of each goal (see the *Measures* section). Progress data were accessible to the authorized health and social care or education professional providing the child with access to Lumi Nova, thereby helping to better inform care and support. Guardians had access to the Lumi Nova webpage, which carried additional psychoeducational information about anxiety.

### Measures

#### Demographic Information

Guardian-completed survey items captured demographic information (age, gender, ethnicity, primary language spoken at home, and eligibility for free school meals) and clinical history (such as previous treatment for anxiety, contact with CAMHS, or a general practitioner or nurse because of anxiety in the previous 3 months) for their child.

#### Anxiety Outcomes

##### Brief SCAS-P-8

The parent-rated brief SCAS-P-8 [12] was used to assess child anxiety symptoms at T1 and T2. The SCAS-P-8 contains 8 items from the original 38-item SCAS [50], which assesses Diagnostic and Statistical Manual of Mental Disorders-fifth edition–related anxiety disorders (generalized anxiety, separation anxiety, social anxiety, panic, and agoraphobia) and is appropriate for use with children aged 7-12 years. Items are scored on a 4-point scale (never, sometimes, often, and always) and summed to derive a total score. Robust psychometric properties have been shown for the SCAS-P-8 [12], and the internal consistency was good (Cronbach *α*=.88) in this sample.

##### RCADS-P Questionnaire

The RCADS-P [51] was used to assess child anxiety and low mood at T1 and T2. The RCADS-P is a 47-item parent report scale comprising 5 subscales that assess symptoms of anxiety diagnoses (separation anxiety disorder, social anxiety disorder, generalized anxiety disorder, panic disorder, and obsessive-compulsive disorder) and one subscale that assesses symptoms of low mood (major depressive disorder). Items are scored on a 4-point scale ranging from 0 to 3 (never, sometimes, often, and always). A total anxiety score (summed anxiety subscale scores; 37 items) and a subscale raw score for major depressive disorder were generated. The RCADS has robust psychometric properties in children and young people [51], and internal consistency for the subscales was good (Cronbach α: range .84-.90) in this sample.

##### CAIS-P Questionnaire

The CAIS-P [52,53] was used to measure the functional impairment of anxiety in children at T1 and T2. The CAIS-P is a 27-item questionnaire that assesses the extent to which anxiety impacts the functioning of children within school, social, and home and family contexts. Two items that were not relevant to preadolescent children (going on a date and having a boyfriend or girlfriend) were excluded. The items are scored on a 4-point scale (score 0 to 3; not at all, just a little, pretty much, and very much) and summed to produce 4 subscales (school, social, home and family, and global) and a total impairment score. The total impairment score was used in this study. The CAIS-P has demonstrated good psychometric properties [52,53], and internal consistency was good (Cronbach α: range .82-.88) in this sample.

##### GBO Tool

The GBO tool [54] was used to measure child-rated progress toward an individual therapeutic goal. Children, supported by a guardian, were asked to select up to 3 goals from 14 common anxiety-related goals prepopulated in Lumi Nova. The available goals were identified by academic clinical partners in relation to common childhood anxieties (eg, for separation anxiety related to being away from a parent or caregiver, “Be able to sleep on their own” was classified as an appropriate goal and for social anxiety, “Be comfortable speaking in front of a group” was deemed an appropriate goal). Children then undertook up to 10 exposure *challenge* steps (in-game and out-of-game challenges with guardian support) to gradually work toward 1 selected goal. Progress toward goal achievement was tracked on a 10-point Likert scale with end points ranging from 0 (no progress toward goal) to 10 (goal reached). GBO scores were collected at T1 and then weekly until a final T2 score was obtained. GBOs are routinely used for outcome monitoring within CAMHS settings and can provide a useful subjective assessment of intervention impact (goal achievement) to support standardized symptom assessment tools.

#### User Engagement and Ease of Use

Anonymized game play data, automatically generated during game play and uploaded to the hub when connected to Wi-Fi, captured game play information, for example, the frequency (total number) of play sessions per player, and duration of play (number of days playing). One question (“How easy is Lumi Nova to play?”) was adapted from the Program Content and Usability questionnaire [55] and assessed child-rated ease of use after the intervention. Scores were rated on a Likert scale ranging from 1 (very easy) to 5 (very hard).

#### Safety

The safety of Lumi Nova was assessed using three indices: (1) change in the major depressive disorder subscale of the RCADS-P (see *Anxiety Outcomes* section) across the intervention; (2) guardian-reported change (positive or negative) in their child at T2, which they attributed to playing Lumi Nova; and (3) guardian-reported adverse events over the duration of the intervention.

#### Open-Response Questions (Optional)

Optional open-response questions for guardians (within the guardian-rated survey at T2) solicited thoughts about the following: (1) guardian perceived changes (positive or negative) associated with playing Lumi Nova, (2) general comment regarding accessing or playing the game, and (3) additional comments. Responses pertinent to the study objectives, that is, those describing (1) effectiveness, (2) user engagement and experience, and (3) safety were summarized.

#### Procedure

All guardians provided informed consent, and the children provided verbal assent before participating in the study. The guardians were asked to complete the demographic and anxiety outcome questionnaires (SCAS-P-8, RCADS-P, and CAIS-P) at T1 using a web-based survey platform. Subsequently, authorized school staff with access to the VitaMind Hub set up child profiles, which automatically triggered an SMS text message to their guardians with access to Lumi Nova via a game key. Participating families were asked to encourage their children to play Lumi Nova multiple times a week over the course of 8 weeks. At the end of the intervention (T2), guardians were asked to complete the anxiety outcome questionnaires (SCAS-P-8, RCADS-P, and CAIS-P).

#### Analytic Strategy

Analyses were performed using SPSS (version 26; IBM Corp). Descriptive statistics were used to summarize sample data; 2-tailed paired sample *t* tests were computed to demonstrate changes in outcome measures before and after the intervention; Wilcoxon signed-rank tests evaluated median difference in goal progression; frequency distributions were computed for ease-of-use scores; and descriptive statistics were used to summarize the game play (duration and frequency of game play sessions), adoption, and completion of exposure challenges in game and in vivo. Simple content analysis summarized and systematized open-response data in accordance with the following study domains identified a priori: effectiveness, user engagement and experience, and safety [56].

## Results

### Anxiety Symptoms and Interference

Mean scores relating to symptom severity and interference before (T1) and after intervention (T2) are reported in Table 2. There was a small reduction in mean scores for symptom severity from T1-T2 for RCADS-P total anxiety and SCAS-P-8. This reduction was statistically significant for SCAS-P-8 (*P*=.009) with a small to moderate effect size and survived correction for multiple analyses. However, no significant difference was reported in RCADS-P total anxiety or in anxiety impairment (CAIS-P).

**Table 2:** Mean change in primary outcome measures for the T1-T2 sample.

Measure		T1, mean (SD)	T2, mean (SD)	*P* value
**Anxiety symptoms**				
	SCAS-P-8^a,b^ (total)	8.33 (4.56)	7.43 (3.28)	.009
	RCADS-P^c^ (total anxiety)	30.73 (13.94)	30.30 (16.92)	.20
**Functional impairment**				
	CAIS-P^d,e^ (total)	20.57 (15.40)	20.97 (15.49)	.80
**Safety**				
	RCADS-P (MDD^f^)	7.07 (4.91)	6.60 (3.94)	.46

^a^SCAS-P-8: Spence Child Anxiety Scale–Parent version.

^b^Only 1 variable (Spence Child Anxiety Scale–Parent version total) was associated with a statistically significant finding (*t*_29_=2.79; *P*=.009; Cohen *d*=0.35), which remained after Bonferroni correction at *P*<.01.

^c^RCADS-P: Revised Child Anxiety and Depression Scale–Parent version.

^d^CAIS-P: Child Anxiety Impact Scale–Parent version.

^e^Significance testing was based on Wilcoxon signed-rank tests for the Child Anxiety Impact Scale–Parent version home and social subscales; otherwise, significance was based on paired sample *t* tests.

^f^MDD: major depressive disorder.

Comparison of the first and last ever child-rated GBO in relation to an active goal established if playing Lumi Nova was associated with therapy-aligned improvement as determined by users. In total, 54 (81%) of the 67 players with game play data selected a goal and subsequently recorded a GBO score for exposure challenges. Out-of-game exposure challenges associated with that goal were recorded for 43 (64%) of the 67 players with game play data, and 45 (67%) players rated their progress by completing at least two GBO scores. A Wilcoxon signed-rank test showed that there was a significant difference between the first and last outcome score over the course of the intervention (*z*=2.433; *P*=.02). On average, players indicated that they had moved closer to reaching their goal over a period of game play, that is, the median score of 7 at the last assessment was significantly higher than the median score of 5 at the first assessment.

Of the 30 guardians who completed the follow-up survey at T2, 16 (53%) provided optional open-response comments. The responses were collated and systematized in relation to the primary study objectives: effectiveness, user engagement and experience, and safety (Table 3).

**Table 3:** Guardian open-response content summarized by research domain (n=16).

Research domain and summarized content			Comments, n (%)
**Effectiveness**			
	Increased confidence and bravery to tackle challenges	6 (38)	
	Increased appreciation that taking small steps is helpful	3 (19)	
	Perceived progression in relation to goal choice	2 (13)	
	Facilitated discussion about anxiety	1 (6)	
	Beneficial in conjunction with other support	1 (6)	
**Engagement and experience**			
	Neutral endorsement of use	5 (31)	
	Laudatory comments	4 (25)	
	Barriers to adoption (design and process)	6 (38)	
	Barriers to adoption (technical barriers)	2 (13)	
	Increased frustration	1 (6)	
**Safety and adverse outcomes**			
	Adverse outcomes	0 (0)	

Regarding effectiveness, comments in this domain were all related to positive improvements in anxiety-related outcomes; 6 (20%) of the 30 guardians described witnessing an increase in confidence or bravery in their child and suggested that children were able to recognize fears and successfully challenge their thoughts:

When she did the challenge, getting an answer wrong, that gave her a bit of confidence that [a] little mistake doesn’t put one in trouble by teachers.

guardian of a girl, aged 12 years

For one child, playing Lumi Nova prompted greater discussion around fears and worries. The child’s guardian said, “He seems more willing to talk about feeling anxious, he asks questions about anxiety” [guardian of a boy, aged 9 years]. Guardians felt that Lumi Nova had generated new learning in line with core processes of exposure therapy about what happens when an anxiety-provoking situation occurs and was effective in helping children work through a step-by step approach:

She liked knowing that she could take small steps towards a recognised fear and liked remembering that she coped with all those steps comfortably.

guardian of a girl, aged 7 years

He took to the game very well and I think it helped him rationalise one of his fears – staying away from home...I definitely think the game put in some excellent groundwork for him to draw on going forward.

guardian of a boy, aged 12 years

In one case, a guardian reported that the game had proved effective in conjunction with existing support: “This, along with weekly play therapy, has helped her anxiety” [guardian of a girl, aged 8 years].

### User Engagement and Experience

Table 4 presents frequency data (average number of game play sessions) over the course of the intervention and duration of game play data (average number of days playing) as an indication of player engagement for those with complete game play data (n=67) and players from the T1-T2 subsample with complete game play data (n=25). Results indicate large variability in the number of times children played the game, ranging from just once to 46 individual episodes of game play out of a maximum potential of 56 episodes, with players averaging 11 (SD 9.41) sessions over a median period of 15 days.

**Table 4:** Average frequency and duration of game play.

			Game play sample (n=67)		T1-T2 sample (n=25)
**Frequency (times played)**					
	Value, mean (SD)	11.22 (9.41)		12.16 (10.45)	
	Value, median (range)	8 (1-46)		8 (1-46)	
**Duration^a^ (days played)**					
	Value, mean (SD)	18.37 (14.75)		18.28 (14.60)	
	Value, median (range)	15 (1-53)		16 (1-53)	

^a^Duration of play from the first recorded date to the last date of game play per participant.

In total, 10 (15%) of the 67 players with game play data rated how easy they found playing Lumi Nova on a scale from 1 (very easy) to 5 (very hard); 8 (12%) players provided a positive or neutral evaluation, with most (6/8, 75%) finding the game easy or very easy, and the rest (2/8, 25%) finding the game neither easy nor hard. Furthermore, 3% (2/67) of players reported finding the game very hard to play.

In total, 18 open-response comments related to player engagement and experience of using Lumi Nova in the T2 guardian survey (Table 3), and 5 neutral comments endorsed the adoption of the game. For example, “Downloaded it and played most days for several weeks” [guardian of a girl, aged 7 years]. A total of 12 comments specifically captured interest in playing the game and its appeal to children. Of these 12 comments, 4 (33%) were laudatory. For example, “[My son] played the game approximately 10 times. He enjoyed it very much...” [guardian of a boy, aged 12 years] and “We’ll miss Lumi Nova...She wanted the chance to deal with other anxieties” [guardian of a girl, aged 7 years]. Six comments suggested that although the premise of the game or elements within it were appealing, there were barriers to its adoption that related to the target audience (a perception it was pitched too young), restrictions in the game processes (eg, limited choice or low relevance of options or insufficient challenge), or a perception of repetition that children found frustrating:

My daughter lost interest in the game and thought it was more aimed at younger children. She has specific worries that weren’t covered.

guardian of a girl, aged 7 years

The feelings bit at the beginning was good, but the tasks following this could be repetitive.

guardian of a boy, aged 9 years

Two guardians commented specifically on technical difficulties (subsequently redressed), which affected the player experience (eg, difficulties downloading the game or saving progress). One guardian simply reported that playing Lumi Nova made her daughter (aged 10 years) *frustrated* but provided no additional context.

### Safety of Lumi Nova

Playing Lumi Nova was not associated with increased symptoms of low mood over the course of the intervention, that is, the mean RCADS-P major depressive disorder scores did not increase from T1 to T2 (Table 2). At T2, 30 parents provided data regarding any positive or negative changes in their child, which were perceived as connected to playing Lumi Nova. In total, of the 30 parents, 22 (73%) reported no change, and the remainder (n=8, 27%) reported positive associated outcomes. No adverse events were spontaneously reported during the course of the intervention. Therefore, overall, there was no evidence to suggest harm or unintended negative consequences associated with playing Lumi Nova.

## Discussion

### Principal Findings

This small-scale preliminary evaluation study examined the effectiveness, user engagement and experience, and safety of Lumi Nova, a mobile app delivering targeted exposure-based CBT strategies for children with mild to moderate difficulties with anxiety. Over an 8-week period of game play, we found that playing Lumi Nova was associated with a reduction in anxiety symptom severity and progress toward treatment goals, and this effectiveness was positively endorsed by guardians. The children engaged with the content and did so safely.

Regarding the app’s effectiveness, there was a reduction in the guardian-rated mean anxiety symptom severity (SCAS-P-8) between T1 and T2 with a small to moderate effect. Such findings are consistent with the literature showing moderate effectiveness in computer-based CBT for childhood anxiety [25,26,36] and contribute to emerging findings from tests on the effectiveness of game-based interventions that have reported moderate child- and parent-rated improvements in symptom severity after a short period of game play [45]. This was a small, low-powered study, and it would be important to establish effectiveness in a larger study. As a simple noncomparative evaluation, we cannot directly attribute symptom severity reduction to Lumi Nova, and the use of an active control group in future studies would help establish whether improvements in anxiety symptomatology could be attributable to the app. Nonetheless, in open responses, guardians associated anxiety-related improvements in children to game play, commenting additionally on perceived broader benefits in relation to increased confidence and successful new learning about stepped approaches to tackling fears and worries.

For player-rated effectiveness, children recorded positive movement toward achieving a self-identified therapy-aligned goal (ie, GBO) over the course of the intervention, on average moving up 2 points toward achieving their goal. Clinically, involving children in the setting and tracking of therapeutic goals provides an essential element of agency and personal activation, which may improve treatment outcomes [30]. Lumi Nova enables players to set a target and chart and reflect on their own progress and demonstrates how the mechanics of a mobile app can facilitate personalization and relevance of treatment. Further exploration of the contribution of this functionality to treatment experience and outcomes would be beneficial. It is noteworthy that this positive child-rated progression contrasts with the parent-reported measurements of effectiveness, which did not support perceived functional improvement in symptom impact. Parent and child informants rating child anxiety symptoms in clinical samples have shown variability in their capacity to identify anxiety disorders [57]. It may also be that parents did not pick up on goal progression in the same way as their child did or that the parent-rated outcome measures were not sufficiently sensitive to this progression. In fact, open-response comments from guardians that identify several positive benefits from participation in their child align with child-reported positive progression. The findings underscore the value of multi-informant approaches in the evaluation of treatment gains. The addition of teacher-rated response measures would offer an additional marker to gauge improvement, particularly where functional impairments manifest within a school context are less apparent at home.

In terms of user engagement and experience, evidence was provided from game play data capturing the quantity of play (frequency and duration of sessions) to indicate game adoption and repeated use over the intervention period. On average, children played Lumi Nova 11 times (SD 9.41) over 18 days (SD 14.75). However, these engagement metrics varied considerably among the players. In addition, data were not reported on the duration of each session of game play, which would help establish that the sessions involved meaningful interaction. In addition to objective (game play) markers, there was also modest support from the limited data that children found the game easy to use. Open-response comments reinforced that children played the game on multiple occasions, sometimes with parents, over many weeks and appeared to enjoy doing so.

It is interesting to note that there is little shared understanding or agreement of what constitutes sufficient engagement for mHealth apps [41] and there is a lack of established usability measures for children [39]. No predefined threshold of sufficient engagement to deliver impact has been specified by the developers of Lumi Nova or targeted in this preliminary study. It is recognized that the optimal dose for intervention effectiveness is likely to vary depending on the user characteristics and context [40]. Notably, 54 (81%) of the 67 players for whom there was game play data selected a goal, completed associated in-game exposure steps and reflections, and recorded at least one GBO score; almost two-thirds (43/67, 64% of players) went on to complete related out-of-game challenges. This engagement with the therapeutic mechanics of the game provides an indicator of engagement breadth and depth [45]. Recently, Zhang et al [58] have suggested that greater understanding of beneficial app interaction for digital health interventions is derived from considering *clinically meaningful activity*, that is, the completion of behaviors indicative of meaningful use (learning, goal-setting, and self-tracking), which is not captured by the *quantity* of engagement. We can gauge user progress in Lumi Nova through in-app progression which is broken down into linear steps. This modular approach is modeled on exposure therapy where each session of use translates to clinically meaningful contact when compared with face-to-face delivery. The receipt of a GBO response thus establishes user progress, as a GBO query event is only triggered when a user has successfully completed all previous steps. Altogether, our findings offer a preliminary indication that Lumi Nova provided an experience that engaged and maintained interest and facilitated progression. However, further work employing inferential analyses which explores how children engage with Lumi Nova (eg, the quantity of play and completion of meaningful activities in game and in vivo) relates to improvements in anxiety symptoms and interference would provide an indication of what might constitute effective and sufficient engagement to deliver treatment benefit [40,41,59].

Gamification is seen as a strategy to increase engagement and adherence with digital mental health interventions by delivering therapeutic content in a format with intrinsic appeal for children [30,33]. To date, few game-based digital mental health interventions specifically developed for children have been empirically evaluated. However, the limited literature that has explored 3D computer and immersive video game approaches for the treatment of anxiety has shown that children enjoy and engage with game-based therapeutic approaches [29] and supports game-based tools to supplement the delivery of therapist-led CBT [44,46]. Lumi Nova’s application of immersive technology and augmented reality to deliver exposure-based CBT strategies in a standalone mobile app is therefore a novel contribution to an emerging evidence base.

Relatively few apps for anxiety in childhood implemented in *real-world* (nontrial) settings have been empirically evaluated [25,26,35]. Promising findings have supported the clinician-supported delivery of CBT skills via smartphones [43]. Our findings extend our understanding of how digital apps can be used to deliver remote self-help interventions and further support the potential of mobile apps to widen reach and facilitate early access to effective treatments for anxiety [26,34]. Given the poor prognosis of anxiety disorders in children when left untreated and the associated burden on health care [60], exploring the potential of digital tools to facilitate and optimize early access to effective treatment and thus prevent the escalation of symptoms and functional impairment is an important focus. Notably, most children recruited to our study did not seek or receive previous treatment for anxiety before starting the intervention, suggesting that participation offered access to evidence-based treatment to a group with an identified need, but for the most part, hidden from services.

Lumi Nova was developed using a robust co-design framework that involved children, parents, teachers, clinicians, academics, and technical experts in prototype design, development, and evaluation via rapid user-testing. This is a strength of the app and in line with guidance, which has called for increased co-design processes that actively engage the intended users and other stakeholders throughout the development cycle of digital game-based innovations for mental health [25,30]. Nonetheless, challenges remain in creating content that maximizes engagement and adherence across a span of ages, disorders, and abilities, which can offer only limited individualization. The ability to respond quickly and modify is an advantage of agile development processes in digital mental health delivery; consequently, many of the learnings identified by children and guardians during this early evaluation (eg, to improve game progression and rewards and cater to a wider range of game play abilities) have now been incorporated. Traditional intervention approaches that assess effectiveness once development is complete diminish the value that can be gained from evaluation during the development process. Digital intervention development enables an iterative multi-cycle approach to improving interventions, codeveloping with users and other stakeholders, as an explicit part of the development process. Rigorous evaluation at an early development phase (as in this study) can improve readiness for product launch. This approach facilitated the achievement of regulatory status (Medicines & Health Care Products Regulatory Agency) for Lumi Nova and its subsequent full market launch.

### Limitations and Future Directions

Although children adopted and engaged with Lumi Nova, and the game play sample was sufficient to demonstrate its use, the evidence of at least one or more sessions of game play was available for only around half of those consenting to play at T1. Analytic information about game play sessions was captured for analysis only when the player’s device had internet connectivity, enabling data to be sent to the data hub, which was not always achieved consistently every week, as directed. Therefore, it is possible that our data underrepresent true player interest and the adoption of the game (ie, game play occurred offline). The drop from those with preintervention consent (n=120) to those with guardians activating an access key (n=74) may have resulted from technical difficulties that guardians faced in downloading the beta version of the game as well as the additional requirement on guardians to complete the SCAS-P-8 to generate the game key. Therefore, poorer uptake may index the study burden on guardians rather than the game’s appeal among players. It would be interesting to analyze adoption and use in a natural (nonstudy) setting. Close partnerships working with teachers and guardians, including practical support with processes of enrollment in the study and game setup, were provided to maximize engagement in the study; nonetheless, guardian retention was a challenge, and this was consistent with other evaluation studies in digital mental health [31]. In addition, the data collection overlapped with the COVID-19 pandemic and the national lockdown in the United Kingdom, which may have had an impact on the study involvement. No data in this study were captured or analyzed from users of the hub (ie, education professionals). It is not clear therefore how users were engaging with the hub and how its functionality, such as access to real-time evidence of player progress, was adopted to support professional decision-making. Of note, the final T1-T2 sample was not sociodemographically diverse and outcomes for this sample may not reflect those that would be obtained (or the appeal more generally for the game) within a broader cross-section of the population.

Further work to establish the maintenance of treatment gains over the short and long term would be an important next step in establishing the effectiveness of Lumi Nova. A study powered to explore potential moderators of effectiveness, engagement, and experience (eg, age, gender, anxiety presentation, additional comorbidities, and disability) would also help clarify who is likely to benefit from playing Lumi Nova and in what circumstances. Contextual factors associated with home-based engagement, such as the level of parental involvement, could be explored [26]. Evidence has shown that parental involvement may play a role in child treatment adherence in CBT [61]. As a remotely delivered digital self-help tool that requires guardian facilitation and supervision, the role of guardian motivation and encouragement to support child engagement with the game remains unclear. As a future direction, it is important to analyze optimum approaches for integrating evidenced digital interventions within care pathways. Work to examine how Lumi Nova sits within and complements the health care ecosystem could, for example, include exploring its clinical use as an adjunct to face-to-face treatment, or where treatment is delayed [26]. Limited health economic data have been reported to support the use of digital health interventions [26,29]. Establishing the cost-effectiveness of Lumi Nova would be an important step in clarifying the value proposition of incorporating a commercially available digital self-help intervention within a clinical implementation model.

### Conclusions

App-based treatment platforms that deliver therapeutic content via gaming technology may provide an opportunity to offer effective early intervention for childhood anxiety disorders and address documented barriers to successful treatment by delivering an appealing and acceptable option for children experiencing difficulties with anxiety that can be accessed within a home environment. This small-scale evaluation study provides early evidence in support of the effectiveness, safety, and acceptability (user engagement and experience) of Lumi Nova, a coproduced and collaboratively developed self-help app delivering exposure-based CBT strategies via immersive technology. Further evaluation is recommended to support and extend these preliminary findings.

## Abbreviations

CAIS-P: Child Anxiety Impact Scale–Parent version
CAMHS: Children and Adolescent Mental Health Service
CBT: cognitive behavioral therapy
GBO: goal-based outcome
mHealth: mobile health
RCADS-P: Revised Child Anxiety and Depression Scale–Parent version
SCAS-P-8: Spence Child Anxiety Scale–Parent version
